# Stability and reproducibility of proteomic profiles measured with an aptamer-based platform

**DOI:** 10.1038/s41598-018-26640-w

**Published:** 2018-05-30

**Authors:** Claire H. Kim, Shelley S. Tworoger, Meir J. Stampfer, Simon T. Dillon, Xuesong Gu, Sherilyn J. Sawyer, Andrew T. Chan, Towia A. Libermann, A. Heather Eliassen

**Affiliations:** 1 Channing Division of Network Medicine, Department of Medicine, Brigham and Women’s Hospital and Harvard Medical School, Boston, MA USA; 2 Department of Epidemiology, Harvard T.H. Chan School of Public Health, Boston, MA USA; 3 Department of Cancer Epidemiology, H. Lee Moffitt Cancer Center and Research Institute, Tampa, FL USA; 4 Department of Nutrition, Harvard T.H. Chan School of Public Health, Boston, MA USA; 5 Genomics, Proteomics, Bioinformatics, and Systems Biology Center and Division of Interdisciplinary Medicine and Biotechnology, Beth Israel Deaconess Medical Center and Harvard Medical School, Boston, MA USA; 6 Cancer Proteomics Core, Dana Farber/Harvard Cancer Center, Boston, MA USA; 7 VA Boston Healthcare System, Boston, MA USA; 8 Division of Gastroenterology, Massachusetts General Hospital and Harvard Medical School, Boston, MA USA

## Abstract

The feasibility of SOMAscan, a multiplex, high sensitivity proteomics platform, for use in studies using archived plasma samples has not yet been assessed. We quantified 1,305 proteins from plasma samples donated by 16 Nurses’ Health Study (NHS) participants, 40 NHSII participants, and 12 local volunteers. We assessed assay reproducibility using coefficients of variation (CV) from duplicate samples and intra-class correlation coefficients (ICC) and Spearman correlation coefficients (r) of samples processed (i.e., centrifuged and aliquoted into separate components) immediately, 24, and 48 hours after collection, as well as those of samples collected from the same individuals 1 year apart. CVs were <20% for 99% of proteins overall and <10% for 92% of proteins in heparin samples compared to 66% for EDTA samples. We observed ICC or Spearman r (comparing immediate vs. 24-hour delayed processing) ≥0.75 for 61% of proteins, with some variation by anticoagulant (56% for heparin and 70% for EDTA) and protein class (ranging from 49% among kinases to 83% among hormones). Within-person stability over 1 year was good (ICC or Spearman r ≥ 0.4) for 91% of proteins. These results demonstrate the feasibility of SOMAscan for analyses of archived plasma samples.

## Introduction

Protein levels in blood can change as a function of age, health, and environmental exposures and can provide important diagnostic, prognostic, and therapeutic information. Therefore, knowing how levels of specific proteins are altered by such factors can be a crucial step toward the understanding of disease etiology and may contribute to disease prevention and treatment. Examples of well-established protein biomarkers include prostate-specific antigen for prostate cancer^1^, hemoglobin A1c for diabetes mellitus^2^, and anti-citrullinated protein antibodies for rheumatoid arthritis^3^. Quantification of valid protein biomarkers in a multiplex setting can be technically challenging, however, because extremely high sensitivity, specificity, and dynamic range are required to profile the human proteome, which consists of an estimated 20,000 proteins as well as splicing and post-translational variants that can differ in concentrations by over 10 orders of magnitude^4^.

High-throughput proteomics technologies have developed rapidly in recent years and have the potential for use in biomarker discovery. One such technology is an aptamer-based proteomics platform called SOMAscan (SomaLogic, Inc, Boulder, Colorado). Aptamers are single-stranded oligonucleotides which fold into molecular structures that bind to proteins with high affinity and specificity in a similar fashion to antibodies^4^. SOMAscan is a highly multiplexed, sensitive platform that uses slow off-rate modified DNA aptamers (SOMAmers) as high affinity protein capture reagents to simultaneously quantify more than 1,300 human proteins in all types of protein extracts, including bodily fluids, tissue, and cells. SOMAscan allows measurement of proteins across a very broad dynamic range, from low abundant cytokines to albumin. SOMAscan has been validated in several studies which have compared proteomic measurements across different platforms^5–11^.

A number of studies published in the last few years have demonstrated the utility of SOMAscan for biomarker discovery. Ganz and colleagues used SOMAscan to quantify proteins in plasma samples to develop and validate a 9-protein risk score for cardiovascular outcomes among patients with stable coronary heart disease^12^. Similarly, Qiao and colleagues investigated the proteomic profile of hepatocellular carcinoma using SOMAscan and identified 68 proteins with differential expression between tumor and non-tumor tissues and 8 proteins associated with vascular invasion^13^. The platform has also been applied to discover biomarkers related to many other diseases including lung cancer^14^, mesothelioma^15^, cancer exosomes^16^, Alzheimer’s disease^17^, influenza^18^, and latent tuberculosis infection^8^.

Despite its potential, this modified aptamer-based proteomics technology has yet to be applied widely to archived blood samples from population-based cohort studies. Because the collection and processing (i.e., centrifuging and aliquotting into plasma, red blood cells, and white blood cells) of blood samples in such studies often do not take place in uniform and ideal conditions, it is critical to know how analytes are influenced by such factors as inter-person differences, blood collection protocol, type of anticoagulant used for collection, and processing delay^19^. Furthermore, within-person reproducibility over time of proteomic profiles is valuable information in prospective studies of diseases with long latency periods that utilize only a single biological sample from each study participant to reflect exposure. Therefore, we conducted the present analysis to examine the feasibility of the SOMAscan platform in studies using archived blood samples by assessing the reproducibility of the assay, effect of delayed processing of samples, and within-person stability over time. We also performed secondary analyses of variations in protein levels by participants’ age, BMI, and fasting status, and compared our results with those from previous reports.

## Methods

### Study Population

The blood samples used in this pilot study included plasma from 16 participants of the Nurses’ Health Study (NHS), 40 participants of the NHSII Mind Body Study (MBS), and 12 local volunteers. The NHS participants were randomly selected from each of three BMI groups (<25, ≥25 to <30, or ≥30 kg/m^2^) in order to assess the potential association between protein levels and BMI. Plasma samples from local volunteers were used to assess assay reproducibility and to compare results by anticoagulant type and sample processing delay. The NHSII MBS samples were used to assess within person-stability over a 1-year period.

The NHS began in 1976 with 121,700 female registered nurses between the ages of 30 and 55 years who responded to a mailed questionnaire. Of these, 32,826 participants donated their blood samples in 1989–1990. Details about the blood collection methods have been reported previously^20,21^. Briefly, each participant collected her blood in two 10 mL sodium heparin tubes and shipped them in a Styrofoam container with an icepack to our laboratory via overnight courier. We received 97% of the samples within 26 hours of blood collection.

The NHSII was established in 1989 with 116,429 female registered nurses aged 25 to 42 years. The MBS is a sub-study among NHSII participants^22^. One of the goals of the MBS is to use repeated biological sample collections to evaluate how specific biomarkers fluctuate over time. In the summer of 2013, 226 MBS participants collected samples of their hair, toenail, saliva, urine, and blood and shipped them to our laboratory via overnight courier. One year later, 208 of the same participants repeated the sample collection procedure. Upon arrival, all samples in the NHS and NHSII MBS collection were centrifuged and separated into plasma, red blood cells, and white blood cells and stored at −130 °C or below in liquid nitrogen freezers.

Volunteers were recruited using fliers hung around our laboratory and local neighborhoods. They were screened for willingness by phone and given an appointment time. At the time of blood draw, all volunteers were consented and asked for basic information including age, sex, race/ethnicity, fasting status, smoking status, multivitamin use, contraceptive use, and pregnancy and breast feeding status. Samples were de-identified at the time of draw with links between identifiable donor information and samples permanently broken. Blood samples from five local volunteers were collected in sodium heparin tubes, those from another five volunteers were collected in EDTA tubes, and those from two volunteers were collected in both types of anticoagulants. Each sample was subsequently separated into four aliquots, of which one was processed immediately, two were processed 24 hours later, and the last one was processed 48 hours after collection. All blood samples were stored in the same manner as described above for the NHS and NHSII samples.

The Institutional Review Board of Brigham and Women’s Hospital approved this study. All research was performed in accordance with relevant guidelines and regulations. Informed consent among the NHS participants was implied by receipt of completed questionnaires and blood samples; written informed consent was obtained from all MBS participants and local volunteers.

### Proteomics Assay

Proteomic profiling was performed using the SOMAscan platform based at the Cancer Proteomics Core of Dana-Farber/Harvard Cancer Center (Boston, MA). SOMAscan utilizes single stranded DNA-based protein affinity reagents called SOMAmers (Slow Off-rate Modified Aptamers), which are discovered *in vitro* through the SELEX (Systematic Evolution of Ligands by EXponential enrichment) aptamer selection technology^4,23–25^. The technology incorporates chemically modified nucleotides that mimic amino acid side chains, resulting in a large variety of aptamers with high specificity and affinity (K_d_ < 1 nM) for their targets^23,26^. The assay transforms the quantity of each protein to be measured into a proportional quantity of a specific SOMAmer, such that the end result is a complex mixture of SOMAmers representative of the proteins that were present in the original sample. Because SOMAmers are DNA molecules, this resulting mixture can be quantified by standard DNA detection techniques such as hybridization to a DNA microarray using relative fluorescence units (RFU) as the readout^27^.

To assay human plasma, the Human Plasma SOMAscan 1.3k kit (SL Part Number 900-00011) was used following the manufacturer’s recommended protocol. Plasma samples (50 μL) from local volunteers and those from NHS participants were assayed together in one batch and samples from NHSII participants were assayed in a second batch. To avoid bias, laboratory personnel were blinded to sample identity, and samples were randomly arranged on the plates, with a set of calibration and normalization samples. Intra-run normalization and inter-run calibration were performed according to SOMAscan assay data quality-control procedures as defined in the SomaLogic good laboratory practice quality system. Data from all samples passed quality-control criteria and were fit for analysis.

### Study Design

This pilot study consisted of three parts: a split replicate pilot, a delayed processing pilot, and a within-person stability pilot. Detailed information about the characteristics of plasma samples included in the study is shown in Table 1. The split replicate pilot was performed to assess assay reproducibility using 14 sets of replicate samples donated by 12 local volunteers, all of which were processed 24 hours after collection. The delayed processing pilot included 36 samples from the same 12 individuals, processed at three different time points—0, 24, or 48 hours after collection. The within-person stability pilot included 80 samples from 40 participants whose blood samples were collected at baseline and again after one year; six blinded quality control samples from two local volunteers were also included. The laboratory was blinded to samples from the same person for all pilots.

**Table 1:** Characteristics of study participants and their plasma samples included in proteomic platform pilot studies.

	Local volunteers (*n* = 12)	NHS (*n* = 16)	NHS II (*n* = 40)
**Study Sample Characteristics, N**			
Total samples collected	56	16	80
Anticoagulant			
EDTA	28	0	0
Heparin	28	16	80
Split replicates	28	0	0
Processing delay after blood collection			
No delay	12	0	0
24-hour delay	24	16	80
48-hour delay	12	0	0
Blood collections over time			
Initial	56	16	40
Follow-up (1 year after initial)	0	0	40
**Study Participant Characteristics** ^**a**^ **, N(%)**			
Mean age, years (range)	39 (24–57)	56 (43–68)	60 (53–65)
Female	9 (75.0)	16 (100.0)	40 (100.0)
Time since last meal			
<8 hours	8 (66.7)	5 (31.3)	6 (15.0)
≥8 hours	4 (33.3)	11 (68.8)	33 (82.5)
Unknown			1 (2.5)
BMI^b^			
<25 kg/m^2^		5 (31.3)	16 (40.0)
≥25 to <30 kg/m^2^		6 (37.5)	13 (32.5)
≥30 kg/m^2^		5 (31.3)	11 (27.5)

^a^For NHS II study participants, characteristics reported in first blood draw.

^b^BMI information was not collected from local volunteers.

### Statistical Analysis

Assay reproducibility and delayed processing stability were examined overall, by anticoagulant type, and by protein class. Within-person stability over time was examined overall and by protein class; analysis by anticoagulant type was not possible because all samples were collected in sodium heparin tubes. We used information obtained from UniProt (www.uniprot.org) to categorize the 1,305 proteins quantified by the SOMAscan assay into nine molecular classes—cytokines (N = 138), growth factors (N = 79), kinases (N = 148), receptors (N = 258), proteases (N = 168), protease inhibitors (N = 45), hormones (N = 35), structural proteins (N = 60), and other/unclassified (N = 499). There were 117 proteins included in more than one category. For example, interleukin (IL)-5 and IL-8 were classified as both cytokines and growth factors.

We observed significant clustering of protein levels (i.e., normalized RFU signal values) by batch in principal components analysis. Although biologic variability across the samples in the two batches may have contributed, significant batch effects were observed even among samples from the same two individuals that were included in both batches, which indicated lab variability. Therefore, we adjusted for batch effects using the sva package in R and carried out analyses of data from both assays combined using the batch-effect-adjusted data set. Within the sva package, the ComBat function directly models and removes known batch effects using empirical Bayes methods^28,29^. Protein levels were natural log transformed prior to batch effects adjustment to improve the normality of protein level distributions. In our present analysis, adjusting for batch effects had minimal impact on the overall results; however, it may make a difference in larger datasets as might be typical in an analysis of proteomics and disease.

Assay reproducibility was quantified using data from the split replicate samples (all of which were processed 24 hours after collection) and calculating the coefficient of variation (CV) for each participant by dividing the standard deviation by the mean protein level and multiplying by 100. Mean CVs were obtained by averaging the CVs across individuals for each protein. We also calculated the percentages of proteins with mean CVs < 20% and those with mean CVs < 10%. In general, CVs < 20% are considered acceptable and CVs < 10% are considered very good with respect to laboratory error^30^. In addition, we calculated Spearman correlation coefficients of mean CVs with mean protein levels across the replicates to assess whether assay reproducibility was associated with protein level.

The effects of processing delay on proteomic profiling were assessed by calculating Spearman correlation coefficients and intraclass correlation coefficients (ICCs). Because we assayed two replicates for samples processed 24 hours after collection, we used the average of the duplicate samples in the analysis. Spearman correlation coefficients were used to compare rankings of protein levels in samples processed immediately versus 24 or 48 hours after collection. ICCs, defined as the between-person variance divided by the sum of the between- and within-person variances, were used to assess the impact of within-person variation relative to the total variation, with higher ICCs representing better stability with delayed processing. To obtain an accurate estimate of between-person variability for calculations of ICCs, data from NHS and NHSII (baseline collection only) participants were also included. Stability with delayed processing is generally considered excellent if the ICC or Spearman correlation coefficient is ≥0.75^30^.

We also examined within-person stability of protein levels over 1 year by calculating Spearman correlation coefficients and ICCs. Data from local volunteers and NHS participants were included in the calculations for between-person variability. Within-person reproducibility over time was considered acceptable if the ICC or Spearman correlation coefficient was ≥0.4^30^.

We conducted stratified analyses of mean protein levels in NHS, NHSII, and volunteer samples processed 24 hours after collection by age (<50, 50–59, or ≥60 years old), fasting status (<8 or ≥8 hours), and BMI (<25, ≥25 to <30, or ≥30 kg/m^2^) by calculating percentage differences in mean protein levels and identifying individual proteins that differed most by these factors. Each protein was evaluated for its associations with fasting, age, and BMI using a robust variance linear regression model. All models were adjusted for age (continuous) and fasting status; BMI was not adjusted for because this variable was not collected from the local volunteers. We then calculated the covariate-adjusted geometric mean protein levels in each category by taking the anti-log of the marginal mean and calculated the percentage difference compared with the referent group. The percentage of proteins with means within 15% of the referent groups’ mean was used as a measure of variability. We repeated the stratified analyses after subsetting the proteins to those that passed our pre-established quality control (QC) criteria of CV < 20% and ICC or Spearman correlation (immediate vs. 24-hour delayed processing) ≥0.75. We applied the Kruskall-Wallis H test on this subset to identify proteins that were expressed differentially (P < 0.01) by age (<50 vs. 50–60 vs. ≥60 years) and BMI (<25 vs. 25–30 vs. ≥30 kg/m^2^) and the Mann-Whitney U test to identify those expressed differentially by fasting status (<8 vs. ≥8 hours). We also applied a false discovery rate correction of P < 0.05 to mitigate the likelihood of obtaining false positive findings due to multiple comparisons^31^.

In a secondary analysis, we compared the within-person difference in mean protein levels by anticoagulant type using data from the two participants whose plasma samples were collected in both types of anticoagulants. Percentage difference was calculated by subtracting the protein levels in EDTA samples from the protein levels in heparin samples and dividing the difference by the protein levels in the EDTA samples, multiplied by 100. All statistical analyses were performed in R version 3.4.0, except for the robust variance linear regression, which was performed using PROC MIXED in SAS version 9.4.

### Data availability

The datasets generated and analyzed during the current study are available from the corresponding author on reasonable request.

## Results

Characteristics of study participants are presented in Table 1. Local volunteers included both men and women, and tended to be considerably younger than NHS and NHSII participants. Among all participants, 72% had fasted for ≥8 hours at the time of blood collection.

**Figure 1:**
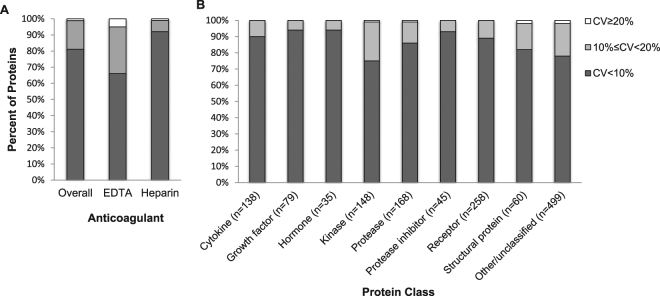
Split replicates’ mean coefficients of variation. Histograms of the percentage of proteins with mean CV < 10%, ≥10% to <20%, and 20% (**A**) overall and by anticoagulant type; (**B**) by protein class.

Figure 1 shows the percentages of proteins with mean CV < 10%, 10% ≤ CV < 20%, and CV ≥ 20% overall and by anticoagulant (A) and by protein class (B). Overall, 99% of protein analytes had CV < 20% and 83% had CV < 10%. Higher proportions of proteins in heparin samples compared with those in EDTA samples had CV < 20% (99% vs. 95%) or CV < 10% (92% vs. 66%). At least 98% of the proteins in each protein class had CV < 20%. The proportions of proteins with CV < 10% ranged from 75% for kinases to 94% for growth factors and hormones. CVs were not significantly correlated with mean protein levels (Spearman r = 0.13).

The ICCs and Spearman r for delayed processing of 24 or 48 hours are shown in Table 2. Among all proteins, 61% had ICC or Spearman r ≥ 0.75 comparing samples processed immediately versus 24 hours after collection. The correlation between samples processed immediately versus 48 hours after collection were generally slightly lower than the correlation between samples processed immediately versus 24 hours after collection (Spearman r ≥0.75, 51% vs. 57%, respectively). The proportion of proteins with ICCs or Spearman r (immediate vs. 24-hour delayed processing) ≥0.75 was higher among EDTA samples (70%) than heparin samples (56%) and varied by protein class, ranging from 49% among kinases to 83% among hormones. In total, there were 796 proteins that passed our pre-established QC criteria of CV < 20% and ICC or Spearman r (immediate vs. 24-hour delayed processing) ≥0.75.

**Table 2:** Intra-class and Spearman correlation coefficients for delayed processing by anticoagulant type and protein class.

	Proteins, N	ICC (0 vs. 24 vs. 48 hr)		Spearman r (0 vs. 24 hr)		Spearman r (0 vs. 48 hr)		ICC or Spearman r (0 vs. 24 hr) ≥0.75, %
		Median (10^th^, 90^th^ percentile)	ICC ≥ 0.75, %	Median (10^th^, 90^th^ percentile)	r ≥ 0.75, %	Median (10^th^, 90^th^ percentile)	r ≥ 0.75, %	
Overall	1,305	0.61 (0.00, 0.92)	37	0.80 (0.27, 0.94)	57	0.75 (0.00, 0.93)	51	61
Anticoagulant								
EDTA	1,305	0.71 (0.00, 0.95)	46	0.75 (0.32, 0.96)	54	0.68 (0.04, 0.93)	42	70
Heparin	1,305	0.60 (0.00, 0.93)	37	0.64 (−0.18, 0.96)	42	0.61 (−0.29, 0.93)	37	56
Protein Class								
Cytokine	138	0.72 (0.00, 0.92)	45	0.84 (0.42, 0.95)	70	0.80 (0.19, 0.94)	62	72
Growth factor	79	0.74 (0.00, 0.93)	48	0.82 (0.20, 0.94)	58	0.79 (0.26, 0.94)	58	65
Hormone	35	0.76 (0.31, 0.95)	51	0.90 (0.68, 0.97)	80	0.83 (0.54, 0.95)	74	83
Kinase	148	0.41 (0.00, 0.92)	31	0.72 (0.13, 0.92)	46	0.64 (−0.23, 0.89)	39	49
Protease	168	0.64 (0.00, 0.90)	38	0.81 (0.33, 0.94)	60	0.76 (0.20, 0.93)	54	67
Protease inhibitor	45	0.60 (0.00, 0.90)	33	0.80 (0.44, 0.95)	51	0.76 (0.40, 0.94)	53	56
Receptor	258	0.66 (0.00, 0.93)	41	0.82 (0.41, 0.94)	64	0.78 (0.25, 0.94)	55	69
Structural protein	60	0.67 (0.00, 0.88)	37	0.80 (0.31, 0.93)	57	0.73 (0.08, 0.92)	48	60
Other/unclassified	499	0.52 (0.00, 0.90)	31	0.75 (0.23, 0.93)	50	0.70 (−0.10, 0.92)	45	54

Within-person stability over a 1-year period was very high overall, with 91% of proteins having ICC or Spearman r ≥ 0.4 and 72% of proteins having ICC or Spearman r ≥ 0.6 (Table 3). Among the 796 proteins that passed our QC criteria, 96% had an ICC or Spearman r ≥ 0.4 and 84% had an ICC or Spearman r ≥ 0.6 (Supplementary Table S1). Across protein classes, the percentage of proteins with ICC or Spearman r ≥ 0.4 ranged from 89% among protease inhibitors to 97% among cytokines and hormones; the percentage with ICC or Spearman r ≥ 0.6 ranged from 65% among kinases to 82% among growth factors and receptors. Results for individual proteins (including mean CVs from the split replicate pilot, ICCs and Spearman r from the delayed processing pilot, and ICCs and Spearman r from the within-person stability pilot) are provided in Supplementary Table S1.

**Table 3:** Intra-class and Spearman correlation coefficients for within-person stability over 1-year period by protein class.

	Proteins, N	ICC		Spearman r		ICC or Spearman r ≥ 0.4, %	ICC or Spearman r ≥ 0.6, %
		Median (10^th^, 90^th^ percentile)	ICC ≥ 0.4, %	Median (10^th^, 90^th^ percentile)	r ≥ 0.4, %		
Overall	1,305	0.70 (0.33, 0.94)	87	0.68 (0.36, 0.87)	87	91	72
Protein Class							
Cytokine	138	0.76 (0.51, 0.93)	95	0.70 (0.45, 0.87)	94	97	80
Growth factor	79	0.78 (0.40, 0.94)	90	0.72 (0.40, 0.85)	90	92	82
Hormone	35	0.75 (0.46, 0.93)	91	0.71 (0.44, 0.91)	94	97	80
Kinase	148	0.68 (0.34, 0.94)	86	0.64 (0.32, 0.84)	86	91	65
Protease	168	0.70 (0.33, 0.92)	85	0.70 (0.39, 0.85)	89	92	75
Protease inhibitor	45	0.72 (0.27, 0.89)	87	0.74 (0.42, 0.87)	89	89	78
Receptor	258	0.77 (0.41, 0.96)	91	0.72 (0.44, 0.89)	93	95	82
Structural protein	60	0.68 (0.42, 0.92)	92	0.67 (0.40, 0.87)	90	93	70
Other/unclassified	499	0.64 (0.23, 0.93)	82	0.65 (0.26, 0.86)	82	86	66

Overall, the median absolute percentage differences in geometric mean protein levels were <5% when comparing each category of age, fasting status, and BMI to the referent group (Table 4). The majority of proteins had mean levels within 15% of the referent group. Similar results were observed when the analyses were restricted to the proteins that passed our QC criteria. Among the study participants whose BMI data were available, adjusting for BMI did not influence the results of mean protein levels by age and fasting status (results not shown).

**Table 4:** Difference in geometric mean protein RFU signal values by age, fasting status, and body mass index.

	Persons, N	All proteins (N = 1,305)		Proteins with CV < 20% and ICC or Spearman r (0 vs. 24 hr) ≥0.75 (N = 796)	
		Median % difference (10^th^, 90^th^ percentiles)	% within ±15% of referent	Median % difference (10^th^, 90^th^ percentiles)	% within ±15% of referent
Age (years)^a^					
<50	14	Referent	Referent	Referent	Referent
50–59	22	−3.7 (−17.6, 14.7)	76	−4.1 (−18.0, 16.9)	73
≥60	31	−4.0 (−16.4, 13.3)	79	−3.5 (−16.2, 16.3)	78
Fasting time (hours)^b^					
<8	19	Referent	Referent	Referent	Referent
≥8	48	−1.7 (−13.0, 8.1)	89	−3.4 (−16.1, 7.5)	84
BMI (kg/m^2^)^c^					
<25	20	Referent	Referent	Referent	Referent
≥25 to <30	19	1.1 (−11.9, 14.1)	85	1.2 (−13.5, 17.2)	80
≥30	16	−1.7 (−18.2, 17.9)	71	0.4 (−16.6, 23.9)	71

^a^Adjusted for fasting time (<8, ≥8 hours).

^b^Adjusted for age (years, continuous).

^c^Adjusted for age (years, continuous) and fasting time (<8, ≥8 hours).

Heatmaps of proteins whose abundance varied the most (Mann-Whitney U test or Kruskall-Wallis H test P < 0.01) by age, fasting status, and BMI are shown in Supplementary Figs S1–S3. After applying a false discovery rate correction of P < 0.05, there were no proteins which differed significantly by age group or fasting status but five proteins which differed by BMI—leptin, fatty acid-binding protein (FABP), C-reactive protein (CRP), interleukin-1 receptor antagonist protein (IL-1ra), and sex hormone-binding globulin (SHBG).

Within the plasma samples from the two local volunteers whose blood was collected using both heparin and EDTA, protein levels were generally lower in the heparin samples than in the EDTA samples at all three processing time points (Supplementary Table S2).

## Discussion

The SOMAscan proteomic platform yielded excellent assay reproducibility and the majority of the protein analytes remained stable over processing delays of up to 48 hours, with some variations by protein class. Most of the proteins also remained stable within the same individuals after one year, with minimal variations by protein class. Our findings of high assay reproducibility of the SOMAscan assay on split samples are similar to those reported by other groups^4,10,32^. To our knowledge, this is the first systematic and comprehensive published report on the SOMAscan 1.3k platform’s reproducibility over sample processing delays and within-person stability. One prior publication evaluated 498 proteins in an early version of SOMAscan (with most of these proteins included in version 1.3 k) for protein stability based on processing delays using a different study design from ours^33^. However, EDTA plasma and serum were analyzed from only four volunteers, and no analysis of heparin plasma was performed. Moreover, processing delays were only analyzed up to 20 hours with either 4 hours or 6 hours at room temperature and the remainder of the 20-hour processing delay at 4 °C. While the specific analytic process in this publication significantly varies from ours, the overall conclusions are consistent with our findings, with ~83% of the proteins being stable up to 20 hours of processing delay.

For most proteins measured by this platform, short (24–48 hour) delays in sample processing did not have a significant impact on the results of the biomarker, supporting use in studies in which samples cannot be processed immediately after collection. In our previous analyses of stability of proteins after 24 or 48 hours of delayed processing using singleplex methods, we reported concentrations of follicular stimulating hormone (FSH), prolactin, sex-hormone binding globulin, Apolipoprotein (Apo) A-1, and Apo B in cooled blood were very stable, with changes ≤1.5% per day^34^. These proteins were also observed to be stable in the present study, with all proteins having ICC or Spearman r (immediate vs. 24-hours delayed processing) ≥0.75 (Supplementary Table S1). In another previous study, we reported that adiponectin levels measured by competitive radioimmunoassay were not significantly different in blood specimens analyzed after 24 or 36 hours of blood collection compared with those which were processed immediately, with an overall ICC of 0.85^35^. This was in agreement with our present study’s observations for adiponectin—overall ICC of samples processed immediately, 24, and 48 hours after blood collection was 0.94 (results not shown). On the other hand, some proteins, such as insulin-like growth factor (IGF-1), had low ICCs due to low between-person variance but acceptable Spearman r values (Supplementary Table S1). Further, the choice of anticoagulant for sample collection may depend on the proteins of interest, as previous studies (using other platforms) as well as our own have also observed somewhat different proteomic profiles using different anticoagulants^36–39^.

Our results also indicate that, for most of the proteins on the SOMAscan platform, a single measurement can reliably estimate average levels over a 1-year period. Among the 796 proteins with acceptable assay reproducibility and delayed processing stability, only 30 showed low within-person stability (i.e., ICC or Spearman r < 0.4). Although we observed high levels of within-person stability even among most of the proteins with poor delayed processing stability, such proteins should be excluded from subsequent longitudinal analyses given the protein concentrations after delay do not adequately reflect circulating concentrations. In our previous study of within-person reproducibility over 1 to 2 years of biomarkers measured with a commercially available ELISA^40^, the ICC was 0.82 for soluble leptin receptor, 0.74 for resistin, 0.91 for matrix metalloproteinase (MMP)-1, 0.69 for MMP2, 0.52 for MMP3, 0.78 for MMP7, and 0.07 for MMP9, which are comparable to the ICCs observed in our present analysis—0.97 for soluble leptin receptor, 0.82 for resistin, 0.87 for MMP1, 0.51 for MMP2, 0.84 for MMP3, 0.86 for MMP7, and 0.36 for MMP9 (Supplementary Table S1). The level of reproducibility we observed for most of the proteins (i.e., ICC or Spearman r ≥ 0.6) is similar to that found for other biological variables such as blood pressure (ICC = 0.6)^41^, blood glucose (ICC = 0.52)^42^, serum cholesterol (ICC = 0.65)^43^, and plasma prolactin (ICC = 0.46 to 0.64)^44^, all exposures considered to be reasonably well-measured and consistent predictors of disease. The general stability of proteomic profiles within individuals suggests that a single blood sample is adequate to represent an individual’s longer-term exposure for most proteins. The overall stability also suggests that alterations of disease-specific sets of proteins may be detectable by comparing longitudinal samples within an individual and could be applied for monitoring disease development or progression.

Overall, mean protein levels were not significantly different by age, BMI, or fasting status, although power was limited by our relatively small sample size. However, it is important to determine whether we observed similar directions of association for biomarkers that have previously been observed to be associated with age, BMI and fasting status as this can provide indirect data on the validity of the platform. For example, previous studies have reported serum insulin-like growth factor binding protein (IGFBP)-5 levels decrease with age whereas IGFBP-4 and FSH levels increase with age^45–49^. In our data, IGFBP-5 decreased with age and FSH increased with age, while IGFBP-4 levels did not vary. For BMI, we observed that CRP, sTNF-R1, sTNF-R2, and leptin all were significantly positively associated with BMI. This is consistent with multiple prior studies of these markers^50–61^. We did not observe associations by fasting status with insulin, ghrelin, AgRP, leptin, and leptin receptor, which have been observed in prior studies^62–64^. This discrepancy may be due to the fact that the study participants in those prior studies had fasted for considerably longer periods compared to those in our present analysis. Overall, we observed results consistent with the prior literature for specific biomarker relationships by age and BMI, supporting that this platform has good biomarker validity.

Several previous studies have validated SOMAscan using other orthogonal proteomic platforms, including LC-MS/MS and ELISA^5–11^. In an analysis of human embryonic and mesenchymal stem cells, Billing and colleagues considered 408 proteins measured by SOMAscan, LC-MS/MS, and RNA sequencing and reported that 60% of the SOMAscan results were validated by the other two methods, which was comparable to results obtained when comparing LC-MS/MS and RNA sequencing to each other^5^. Murota and colleagues used SOMAscan to identify 33 biomarkers associated with rheumatoid arthritis and selected five proteins to validate the results using conventional immunoassays, including electrochemiluminescence assays, ELISA, and latex turbidimetric assays^6^. Levels in RFU of SOMAscan were highly correlated with concentrations in conventional assays, with Spearman correlation coefficients ranging between 0.745 and 0.977^6^. In another study, Coenen-Stass *et al*. identified 96 biomarkers associated with Duchenne Muscular Dystrophy in mice using SOMAscan and validated five out of six novel markers using ELISA^7^. The one protein which was not validated was not detectable by ELISA. Moreover, Ngo and colleagues developed an aptamer-based immunoaffinity pull down assay combined with LC-MS/MS that analytically validated the specificity of SOMAscan-derived aptamers for eight plasma proteins^10^.

Our study was limited by the relatively small number of samples, resulting in low precision of our estimates. For the same reason, the between-person variability may be lower than what it would have been in a larger study, suggesting our results for the ICCs may have been underestimated. However, the ICCs were reasonably high for the large majority of the markers. Furthermore, because most of our study participants were white females, we were not able to investigate whether protein levels and their associations with age, fasting, and BMI differed by sex and race.

With the recent advances in proteomics technology, we are now able to collect large amounts of high-quality information regarding the human proteome. The results of our pilot study demonstrate the feasibility of aptamer-based proteomic profiling technology for use in studies where blood samples may not be collected under ideal clinical conditions and may only be collected once from each participant due to cost and logistical reasons. Notably, our results show that this proteomics platform has excellent laboratory reproducibility, minimal effect of delayed sample processing, and good within-person stability over time, and thus could be considered for use even in studies with stored specimens, as is common in many epidemiologic studies. The application of proteomics to biomarker research will allow in-depth investigations of the role of individual proteins and protein classes in disease etiology, and understanding the intersection of the proteome with other aspects of our biological system, such as the genome and metabolome, will bring us closer to the promise of precision medicine.

## Electronic supplementary material

Supplementary Figures and Tables

Supplementary Table S1
