# Supplementary material for: Stability and reproducibility of proteomic profiles measured with an aptamer-based platform
Source: Sci Rep. 2018 May 30;8:8382. doi: 10.1038/s41598-018-26640-w (PMC5976624; doi:10.1038/s41598-018-26640-w)
Supplement: Supplementary file 1 — Supplementary Figures and Tables [file 41598_2018_26640_MOESM1_ESM.docx]

Stability and reproducibility of proteomic profiles measured with an aptamer-based platform

Claire H. Kim, Shelley S. Tworoger, Meir J. Stampfer, Simon T. Dillon, Xuesong Gu, Sherilyn J. Sawyer, Andrew T. Chan, Towia A. Libermann, A. Heather Eliassen

**
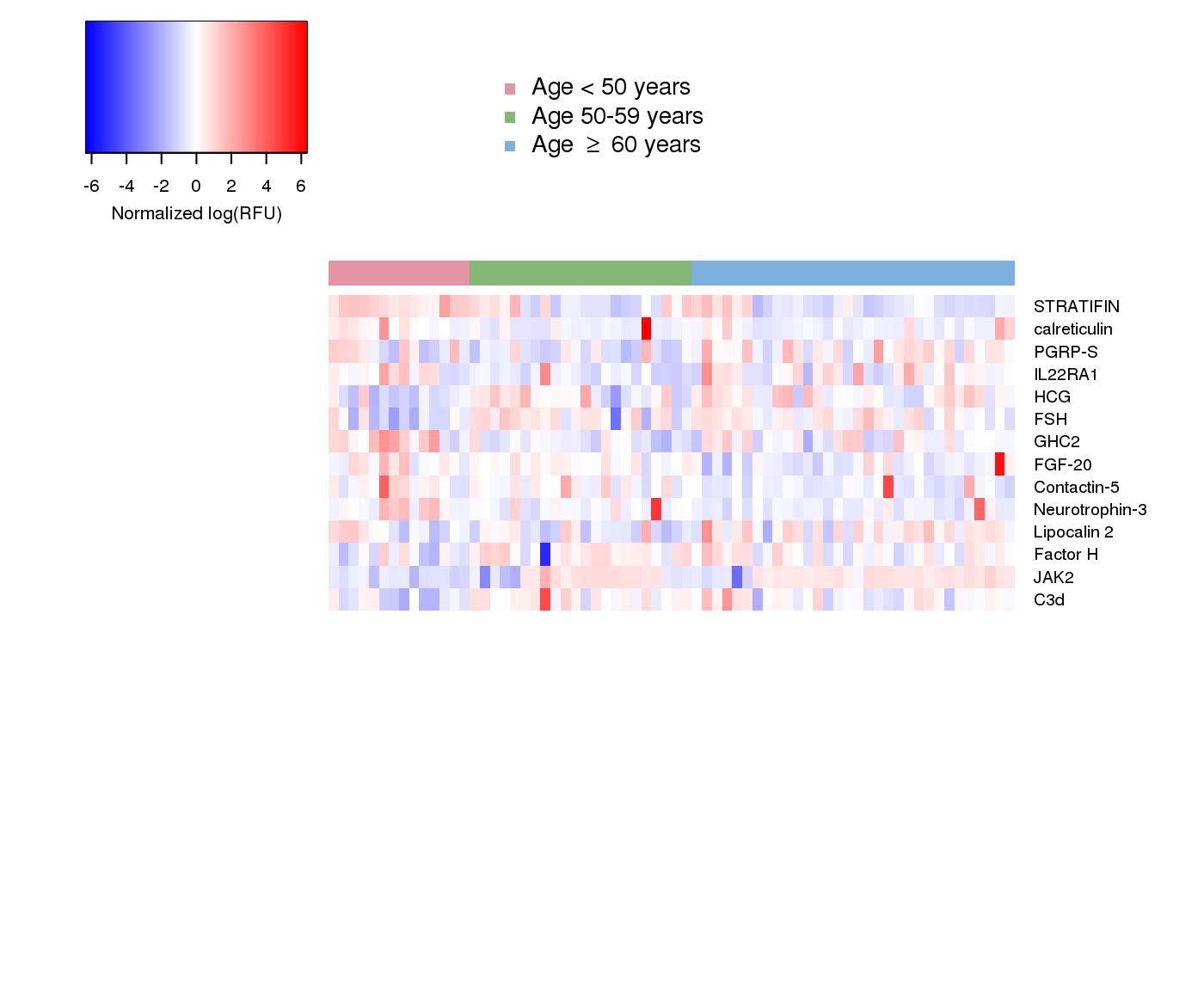
**

| **Protein (UniProt ID)** | **Mean RFU** | | | **Kruskal-Wallis H-test**  **P-value** |
| --- | --- | --- | --- | --- |
|  | **Age <50 years** | **Age 50-59 years** | **Age ≥ 60 years** |  |
| STRATIFIN (P31947) | 2,622 | 1,846 | 1,701 | 0.0001 |
| calreticulin (P27797) | 1,860 | 1,503 | 1,442 | 0.0040 |
| PGRP-S (O75594) | 1,155 | 805 | 1,404 | 0.0042 |
| IL22RA1 (Q8N6P7) | 771 | 609 | 739 | 0.0071 |
| HCG (P01215) | 1,716 | 4,045 | 4798 | 0.0044 |
| FSH (P01225) | 1,041 | 2,400 | 2481 | 0.0040 |
| GHC2 (Q9H1K4) | 408 | 330 | 356 | 0.0012 |
| FGF-20 (Q9NP95) | 379 | 363 | 326 | 0.0012 |
| Contactin-5 (O94779) | 437 | 401 | 353 | 0.0092 |
| Neurotrophin-3 (P20783) | 228 | 189 | 154 | 0.0032 |
| Lipocalin-2 (P80188) | 64,431 | 59,299 | 7,2647 | 0.0084 |
| Factor H (P08603) | 54,301 | 59,644 | 58,561 | 0.0083 |
| JAK2 (O60674) | 17,699 | 34,660 | 39,552 | 0.0003 |
| C3d (P01024) | 11,310 | 16,567 | 14,631 | 0.0040 |

**Supplementary Figure S1. Proteins with the largest difference in mean RFU signal values by age.**

Top: Heatmap of proteins with Kruskal-Wallis H-test P-value <0.01 comparing samples from participants aged <50 vs. 50-59 vs. ≥60 years. Bottom: Mean RFU values and P-values for the proteins in the heatmap.

**
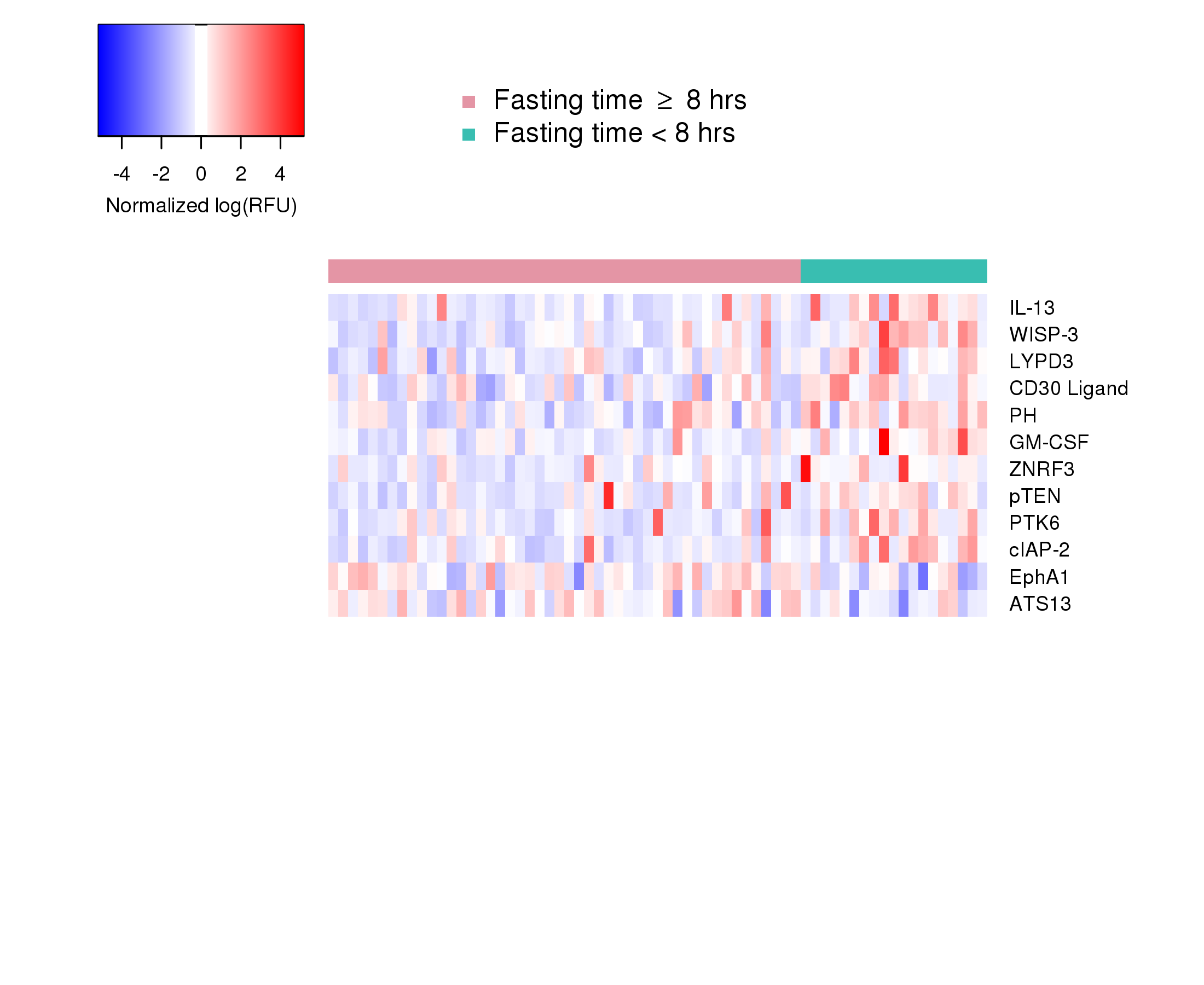
**

| **Protein (UniProt ID)** | **Mean RFU** | | **Mann-Whitney U-test**  **P-value** |
| --- | --- | --- | --- |
|  | **Fasting time ≥8 hours** | **Fasting time <8 hours** |  |
| IL-13 (P35225) | 582 | 805 | 0.0023 |
| WISP-3 (O95389) | 563 | 714 | 0.0011 |
| LYPD3 (O95274) | 848 | 1,033 | 0.0069 |
| CD30 Ligand (P32971) | 1,400 | 1,598 | 0.0082 |
| PH (P01298) | 926 | 1,471 | 0.0006 |
| GM-CSF (P04141) | 311 | 420 | 0.0006 |
| ZNRF3 (Q9ULT6) | 275 | 399 | 0.0062 |
| pTEN (P60484) | 440 | 559 | 0.0065 |
| PTK6 (Q13882) | 167 | 213 | 0.0065 |
| cIAP-2 (Q13489) | 141 | 177 | 0.0024 |
| EphA1 (P21709) | 13,123 | 10,011 | 0.0099 |
| ATS13 (Q76LX8) | 2,892 | 2,540 | 0.0081 |

**Supplementary Figure S2. Proteins with the largest difference in mean RFU signal values by fasting time.**

Top: Heatmap of the proteins with Mann-Whitney U-test P-value <0.01 comparing samples from participants who had fasted ≥8 vs. < 8 hours at the time of blood collection. Bottom: Mean RFU values and P-values for the proteins in the heatmap.

**
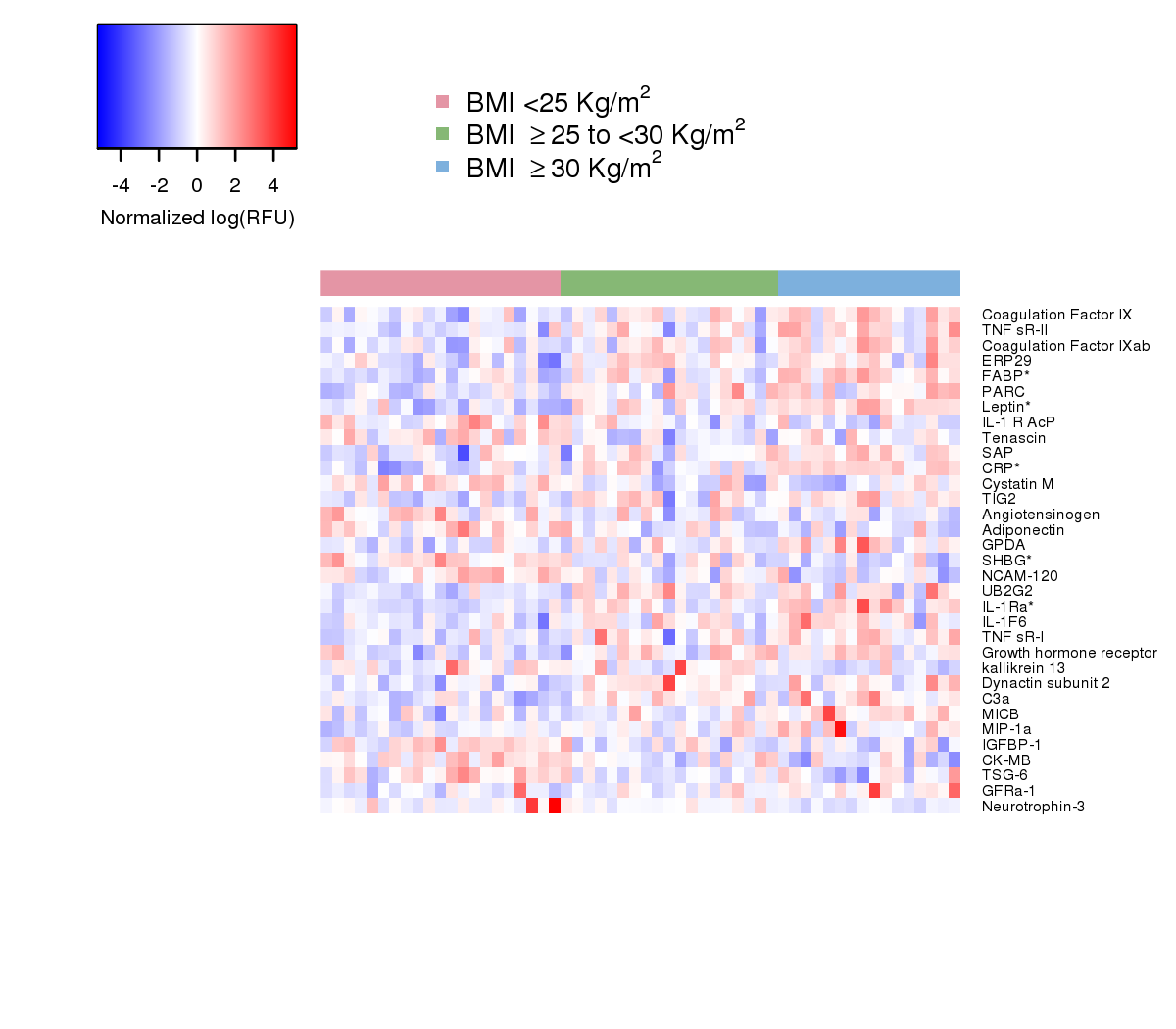
**

| **Protein (UniProt ID)** | **Mean RFU** | | | **Kruskal-Wallis H-test**  **P-value** |
| --- | --- | --- | --- | --- |
|  | **BMI <25 Kg/m^2^** | **BMI ≥25 to <30 Kg/m^2^** | **BMI ≥30Kg/m^2^** |  |
| Coagulation Factor IX | 8,374 | 9,024 | 9,827 | 0.0028 |
| TNF SR-II | 7,195 | 7,830 | 9,300 | 0.0022 |
| Coagulation Factor IXab | 6,642 | 7,113 | 7,744 | 0.0067 |
| ERP29 | 5,496 | 6,468 | 6,684 | 0.0054 |
| FABP* | 6,967 | 8,774 | 13,269 | 4×10^-5^ |
| PARC | 4,960 | 7,455 | 8,325 | 0.0020 |
| Leptin* | 4,259 | 7,576 | 12,998 | 1×10^-5^ |
| IL-1 R AcP | 21,493 | 17,047 | 15,670 | 0.0067 |
| Tenascin | 17,391 | 13,782 | 15,674 | 0.0088 |
| SAP | 25,078 | 27,376 | 30,499 | 0.0074 |
| CRP* | 12,085 | 20,160 | 39,264 | 4×10^-5^ |
| Cystatin M | 3,065 | 2,370 | 2,354 | 0.0006 |
| TIG2 | 2,047 | 2,315 | 2,477 | 0.0017 |
| Angiotensinogen | 3,491 | 2,710 | 2,481 | 0.0056 |
| GPDA | 1,696 | 1,891 | 2,492 | 0.0032 |
| SHBG* | 4,077 | 2,446 | 1,870 | 0.0003 |
| NCAM-120 | 5,808 | 4,928 | 4,528 | 0.0050 |
| UB2G2 | 4,399 | 5,236 | 5,321 | 0.0068 |
| IL-1Ra* | 3,024 | 3,681 | 4,852 | 0.0001 |
| IL-1F6 | 2,434 | 3,165 | 3,857 | 0.0027 |
| TNF sR-1 | 769 | 857 | 968 | 0.0022 |
| Growth hormone receptor | 644 | 806 | 822 | 0.0072 |
| kallikrein 13 | 747 | 757 | 657 | 0.0020 |
| Dynactin subunit 2 | 585 | 695 | 688 | 0.0021 |
| C3a | 491 | 695 | 741 | 0.0022 |
| MICB | 688 | 745 | 1,117 | 0.0033 |
| MIP-1a | 756 | 1,007 | 1,337 | 0.0039 |
| IGFBP-1 | 1,239 | 645 | 598 | 0.0021 |
| CK-MB | 981 | 719 | 526 | 0.0039 |
| TSG-6 | 515 | 402 | 408 | 0.0078 |
| GFRa-1 | 248 | 264 | 341 | 0.0013 |
| Neurotrophin-3 | 202 | 167 | 131 | 0.0030 |

**Supplementary Figure S3. Proteins with the largest difference in mean RFU signal values by BMI.**

Top: Heatmap of the proteins with Kruskal-Wallis H-test P-value <0.01 comparing samples from participants with BMI <25 vs. ≥25 to <30 vs. ≥30 kg/m^2^. Bottom: Mean RFU values and P-values for the proteins in the heatmap.

*FDR-adjusted P-value <0.05

**Supplementary Table S2. Differences in mean protein RFU signal values by anticoagulant type (heparin vs. EDTA)**

|  | **Donor A** | | |  | **Donor B** | | |
| --- | --- | --- | --- | --- | --- | --- | --- |
| **% difference^a^** | **0-hr samples,**  **%** | **24-hr samples,**  **%** | **48-hr samples,**  **%** |  | **0-hr samples,**  **%** | **24-hr samples,**  **%** | **48-hr samples,**  **%** |
| ≤ -75% | 5 | 2 | 2 |  | 2 | 2 | 2 |
| > -75% to -50% | 18 | 12 | 10 |  | 8 | 12 | 11 |
| > -50% to -25% | 28 | 27 | 31 |  | 34 | 28 | 23 |
| > -25% to 0% | 19 | 22 | 32 |  | 33 | 33 | 31 |
| > 0% to 25% | 19 | 19 | 11 |  | 13 | 13 | 14 |
| > 25% to 50% | 7 | 10 | 5 |  | 3 | 5 | 6 |
| > 50% to 75% | 2 | 3 | 3 |  | 2 | 2 | 4 |
| > 75% | 3 | 5 | 6 |  | 4 | 6 | 9 |

^a^ % difference = (protein RFU signal in heparin sample – protein RFU signal in EDTA sample)/protein RFU signal in EDTA sample × 100
